# A Substrate-Mimicking Basement Membrane Drives the Organization of Human Mesenchymal Stromal Cells and Endothelial Cells Into Perivascular Niche-Like Structures

**DOI:** 10.3389/fcell.2021.701842

**Published:** 2021-09-28

**Authors:** Valeria Perugini, Matteo Santin

**Affiliations:** Centre for Regenerative Medicine and Devices, School of Applied Sciences, University of Brighton, Brighton, United Kingdom

**Keywords:** stromal cells, basement membrane (BM), biomaterials-cells, perivascular stromal cells, spheroids, stromal cell niche

## Abstract

Extracellular matrix-derived products (e.g. Matrigel) are widely used for *in vitro* cell cultures both as two-dimensional (2D) substrates and as three-dimensional (3D) encapsulation gels because of their ability to control cell phenotypes through biospecific cues. However, batch-to-batch variations, poor stability, cumbersome handling, and the relatively high costs strictly limit their use. Recently, a new substrate known as PhenoDrive-Y has been used as 2D coating of tissue culture plastic showing to direct the bone marrow mesenchymal stromal cells (MSCs) toward the formation of 3D spheroids. When organized into 3D spheroids, the MSCs expressed levels of pluripotency markers and of paracrine angiogenic activity higher than those of the MSCs adhering as fibroblast-like colonies on tissue culture plastic. The formation of the spheroids was attributed to the properties of this biomaterial that resemble the main features of the basement membrane by mimicking the mesh structure of collagen IV and by presenting the cells with orderly spaced laminin bioligands. In this study, PhenoDrive-Y was compared to Matrigel for its ability to drive the formation of perivascular stem cell niche-like structures in 2D co-culture conditions of human endothelial cells and adult bone marrow MSCs. Morphological analyses demonstrated that, when compared to Matrigel, PhenoDrive-Y led endothelial cells to sprout into a more consolidated tubular network and that the MSCs nestled as compact spheroids above the anastomotic areas of this network resemble more closely the histological features of the perivascular stem cell niche. A study of the expressions of relevant markers led to the identification of the pathways linking the PhenoDrive-Y biomimicking properties to the acquired histological features, demonstrating the enhanced levels of stemness, renewal potential, predisposition to migration, and paracrine activities of the MSCs.

## Introduction

The bone marrow is a source of progenitor cells including the mesenchymal stromal cells (MSCs) (Charbord, 2010). In the bone marrow and in other tissues, MSCs typically reside in perivascular niches supported by the basement membrane (BM) (Putnam, 2014), and their presence seems to be essential for both organ remodeling and repair (Bajek et al., 2011). Beyond their potential to differentiate and renew tissues, MSCs have also shown abilities to secrete angiogenic factors, including vascular endothelial growth factor (VEGF) and platelet-derived growth factor (PDGF), that directly influence the activities of the neighboring vascular endothelial cells (ECs), thus playing a role in both the formation and maturation of blood vessels (Strioga and Michalek, 2012). The biochemical pathways controlling cell interactions and cross-talks at the perivascular niche have not been fully elucidated, but it is reasonable to hypothesize that the BM can play an important role because of its range of interactions with both MSCs and ECs (Potapova and Doronin, 2007). Indeed, the BM is a key component of both stem cell niches and endothelia as it is made of various macromolecules, including collagen IV and laminin, that provide structural support and biochemical stimuli to both cell types (Matsubara and Kato, 2004). More specifically, the BM maintains MSCs in a dormant state ready to differentiate and migrate, and it stimulates them to provide paracrine signaling to neighboring ECs (Kruegel and Miosage, 2010). Upon injury, MSCs are induced to migrate to the damaged area and to differentiate into tissue-specific phenotypes (Sillat et al., 2012). To date, the role of the BM within niches has been studied in *in vitro* cell culture (Benton et al., 2009). Particularly, natural substrates such those derived from BM-producing tumors (i.e., Matrigel^TM^) are widely used for their availability and versatility to various cell types (Hughes et al., 2010). However, batch-to-batch variations, limited stability, and costs are among the recognized drawbacks of these substrates (Kohen et al., 2009). Contrarily, the recent development of synthetic alternatives has shown great promise for MSCs and organotypic cultures (Schmids et al., 2018). Unlike Matrigel, these substrates have been demonstrated to regulate specific cellular pathways such as those controlled by key components of the native BM, including laminin (Hagbard and Kallur, 2018). In particular, biomaterials functionalized with the short laminin peptide sequence YIGSR have demonstrated the ability to modulate several adhesion-related cell activities, including cell cycle and migration, through biospecific interactions with cell surface receptors, i.e., the integrins (Frith and Cooper-White, 2012). Despite the progress achieved using these substrates, the uncontrolled spacing and density of these motifs have currently prevented them to instruct MSCs in assuming an abluminal position akin to their perivascular organization *in vivo* (Ehninger and Trumpp, 2011). Starting from these observations, PhenoDrive-Y, a novel substrate that has been demonstrated to mimic *in vitro* both the biospecific and physical features driving MSC interactions with the BM *in vivo* (Perugini and Santin, 2017), was considered a suitable substrate to study the MSC phenotype and paracrine activities when sitting in the perivascular niche. Previous work has demonstrated that, by combining nanotopography with a precise YIGSR distribution, this substrate leads MSCs to spontaneously form spheroids expressing levels of pluripotency markers relatively higher than those of the 2D spreading and spindle-shaped MSCs that form fibroblast-like colonies on tissue culture plastic (Perugini and Santin, 2020). In the present work, PhenoDrive-Y was used as a coating of standard tissue culture plasticware for the co-culture of MSCs and human umbilical vascular ECs (HUVECs) to induce the formation of perivascular niche-like structures. Both the intracellular pathways and the paracrine activities of the cells were investigated and related to those obtained when the same co-cultures were performed on Matrigel.

## Materials and Methods

### Preparation of Cell Substrates

All experiments were performed on 24-well tissue culture plates (Fisher Scientific, Loughborough, United Kingdom) coated either with a growth factor reduced BM matrix (GFR Matrigel, BD Biosciences, Worthing, United Kingdom) or PhenoDrive-Y (Tissue Click, Hove, United Kingdom) following the manufacturers’ instructions under sterile conditions. PhenoDrive-Y-coated plates underwent an additional sterilization process by UV irradiation at 256 nm wavelength using a UV lamp (Perkins, Peterborough, United Kingdom) for 1 h.

### Cell Culture

HUVECs were purchased from the LGC-ATCC Group (London, United Kingdom) and suspended in serum-free F12-K medium (LGC-ATCC, London, United Kingdom) supplemented with 0.1 mg/ml heparin and 0.05 mg/ml endothelial cell growth supplement (ECGS; Sigma-Aldrich, Gillingham, United Kingdom). HUVECs (cellular passages 11, 14, and 18) were cultured either in monoculture or in co-culture with human MSCs (three female donors aged 21–22 years; Lonza, Slough, United Kingdom) from passage 2 at a 2:1 HUVECs/MSCs seeding ratio and grown in a combined (1:1) F12-K/TheraPEAK^TM^ MSCGM-CD^TM^ chemically defined MSC medium (Lonza, Slough, United Kingdom). In both monoculture and co-culture conditions, the cell seeding density for both Matrigel and PhenoDrive-Y was 100,000 cells/ml (in co-culture, 60,000 HUVEC/40,000 MSCs), at 37°C and 5% CO_2_ for 18 and 48 h.

### Image Analysis

Phase-contrast images of the cells were acquired after 18 and 48 h of culturing using Leica TCS SP5 confocal laser scanning microscope (Leica Microsystems, Heidelberg, Germany) and Nikon Eclipse TE2000-U light-fluorescence microscope (Nikon, Tokyo, Japan) with digital SLR camera with ×10 and ×20 objective lenses. The formation of angiogenic sprouting was evaluated and quantified from these images by measuring the number of (i) tubule-like structures, (ii) anastomosis-like junctions, and (iii) meshes using an ImageJ program, Angiosys 1.0 (TCS Cellworks, Buckingham, England) as previously described (Steinle and Avci-Adali, 2018). Each tube formation assay was performed as three independent experiments, and the data were expressed as the mean ± standard deviation (SD, *n* = 9). Quantification of the relative fluorescence intensity (RFU) was performed with ImageJ software^1^ as previously described (Vergara et al., 2017).

For immunofluorescence staining, the cells were fixed with chilled methanol for 10 min at −20°C and washed twice with phosphate-buffered saline (PBS; Sigma-Aldrich, Gillingham, United Kingdom) before being incubated with PBS-Tween (0.05%, *v*/*v*, Tween-20) and then 1% (*w*/*v*) bovine serum albumin (BSA; Sigma-Aldrich, Gillingham, United Kingdom) for 1 h at room temperature. Both HUVECs and HUVECs/MSCs were incubated with the antibodies anti-human primary CD31, CD90, RhoA, Rac1, CXCR4 (1:100, Abcam, Cambridge, United Kingdom) and HIF-1α (1:50; Abcam, Cambridge, United Kingdom) at 4°C overnight. Later, the samples were incubated with either 488- or 594-nm-detectable fluorophore-conjugated secondary antibodies (1:100; Fisher Scientific, Loughborough, United Kingdom) for 1 h at room temperature, dark conditions, and their nuclei counterstained with 4′, 6-diamidino-2-phenylindole (DAPI; Fisher Scientific, Loughborough, United Kingdom). Images were taken using a confocal microscope (Leica TCS SP5) with ×20 objective lenses. To facilitate the identification of the different cell phenotypes, HUVEC 594-detectable secondary antibody fluorescence was converted into a blue fluorophore by refining the photomultiplier tube (PMT) in the confocal software.

The cell cytoskeleton organization of HUVECs when in direct contact with the two different substrates was studied using rhodamine–phalloidin staining. The cells were fixed by adding 4% (*v*/*v*) paraformaldehyde for 10 min and stained using rhodamine–phalloidin solution (1:100; Sigma-Aldrich, Gillingham, United Kingdom) in PBS for 1 h at room temperature. They were then washed twice in PBS and the cells at the tip of sprouting structures analyzed using a TCS SP5 confocal laser scanning microscope, as reported above.

### Western Blotting

After 48 h incubation, HUVECs and HUVECs/MSCs were scraped off from PhenoDrive-Y, whilst those cultured on Matrigel were detached by treatment with a cell recovery solution (BD Biosciences, Worthing, United Kingdom) for 1 h at 4°C. The cells were lysed for 10 min in lysis RIPA buffer supplemented with 50 μg/ml protease inhibitors (Sigma-Aldrich, Gillingham, United Kingdom) and then centrifuged at 12,000 × *g* for 10 min at 4°C. The protein concentration in the supernatants was measured using Bradford reagent (Sigma-Aldrich, Gillingham, United Kingdom). Afterward, 30 μg from each sample was separated by 10% (*w*/*v*) sodium dodecyl sulphate (SDS)-polyacrylamide gel electrophoresis and transferred onto a nitrocellulose filter membrane (Amersham, Little Chalfont, United Kingdom) at 20 mV overnight. The membranes were treated with 0.01% (*w*/*v*) BSA and incubated with various primary antibodies [β1-integrin (1:500; R&D Systems, Oxford, United Kingdom); pAkt (1:1,000; Abcam, Cambridge, United Kingdom); VEGFR2 (1:1,000; Abcam, Cambridge, United Kingdom); EGFR (1:1,000; Abcam, Cambridge, United Kingdom); PDGFR-β (1:500; Cell Signaling, London, United Kingdom); and H3K27me3 (1:1,000; Cell Signaling, London, United Kingdom)] prior to incubation with horseradish peroxidase (HRP)-conjugated secondary antibodies (1:1,000; Fisher Scientific, Loughborough, United Kingdom) for 1 h at room temperature. An enhanced chemiluminescence (ECL) detection kit (Amersham, Little Chalfont, United Kingdom) was then applied to detect protein bands with intensities related to a positive control, GAPDH, and quantified using ImageJ. Values were normalized with respect to the loading control.

### Statistics

Each experiment was performed at least in triplicate, unless differently specified. Data were expressed as the mean ± SD. Statistical significance (*p* ≤ 0.05) was determined using one-way ANOVA.

## Results

**FIGURE 1:**
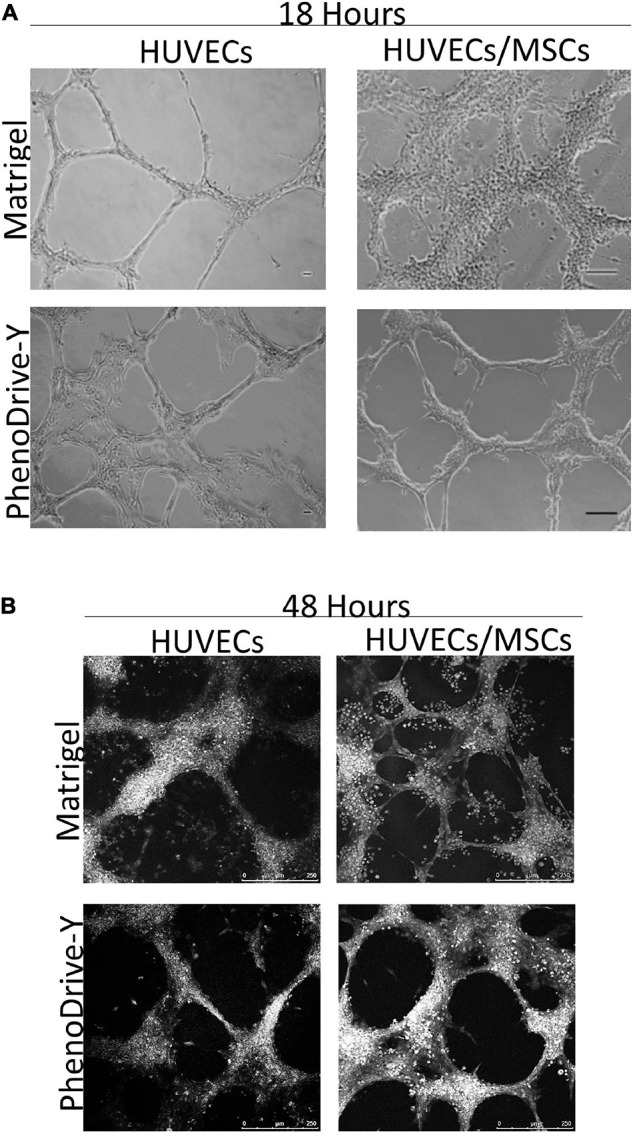
**(A,B)** Tubule formation of human umbilical vascular endothelial cells (HUVECs) and HUVECs/mesenchymal stromal cells (MSCs) grown on Matrigel and PhenoDrive-Y after 18 h **(A)** and 48 h **(B)** incubation. Light microscopy: *scale bar*, 100 μm; RGB bright-field confocal microscopy: *scale bar*, 250 μm.

Light and bright-field microscopy highlighted the formation of angiogenic sprouting when HUVECs were cultured either in monoculture or in HUVEC/MSC co-culture on both substrates (Figure 1). After 18 h of incubation, HUVECs seeded on Matrigel formed a complex network of branching capillary tubules that appeared thinner and discontinued (Figure 1A, HUVECs, top micrograph) compared to those observed on PhenoDrive-Y, where a marked development of an anastomosing tubular network was observed (Figure 1A, HUVECs, bottom micrograph). In the case of co-culture with MSCs on Matrigel, large clusters of cells coalesced toward the endothelial sprouting network to form a rather disorganized pattern partly adhering on top of the sprouting and partly adhering on the substrate surface in proximity of the sprouting (Figure 1A, HUVECs/MSCs, top micrograph). In comparison, cells on PhenoDrive-Y were tightly associated to the tubules to form 3D structures particularly at the areas of anastomosis (Figure 1A, HUVECs/MSCs, bottom micrograph).

At 48 h incubation (Figure 1B), the tubule network, formed by HUVECs monoculture on Matrigel, showed signs of regression that were inhibited when these cells were co-cultured with MSCs on the same substrates. Although the tubules appeared disorganized and disconnected, the presence of round cells was mainly found around the anastomotic areas and in proximity to the HUVEC sprouts (Figure 1B, HUVECs/MSCs, top micrograph). In comparison, PhenoDrive-Y prompted HUVECs to organize themselves into complex, highly branched capillary-like structures that established tight contacts with clusters of cells (Figure 1B, HUVECs/MSCs, bottom micrograph). At both experimental points and in HUVEC monoculture and HUVEC/MSC co-cultures, PhenoDrive-Y ensured a complete coverage of the well bottom, whereas Matrigel showed the absence and regression of the endothelial sprouting particularly in proximity to the periphery of the well (Supplementary Figure 1). MSC monoculture on the type of Matrigel used in this study showed increasing formation of spheroidal MSC clusters (Supplementary Figure 1B).

**FIGURE 2:**
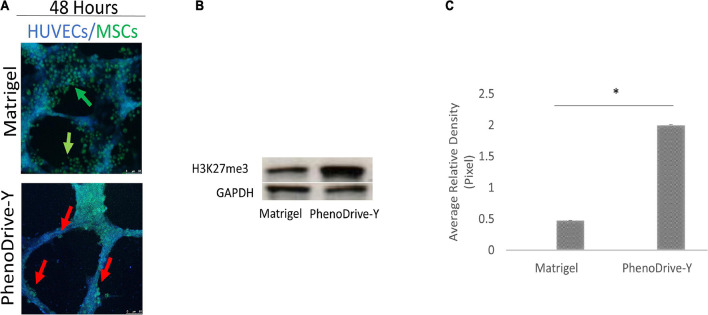
Mesenchymal stromal cell (MSC) organization on both substrates during co-culture conditions after 48 h incubation. **(A)** MSCs were stained with CD90 (*green*). *Scale bar*, 50 μm. **(B)** Western blot analysis of the levels of H3K27me3 compared to the GAPDH loading control. *Green arrows*, HUVEC tubules, *red arrows*, individual, and clusters of MSCs. **(C)** Quantification of H3K27me3 expression by image analysis; *p < 0.01.

**FIGURE 3:**
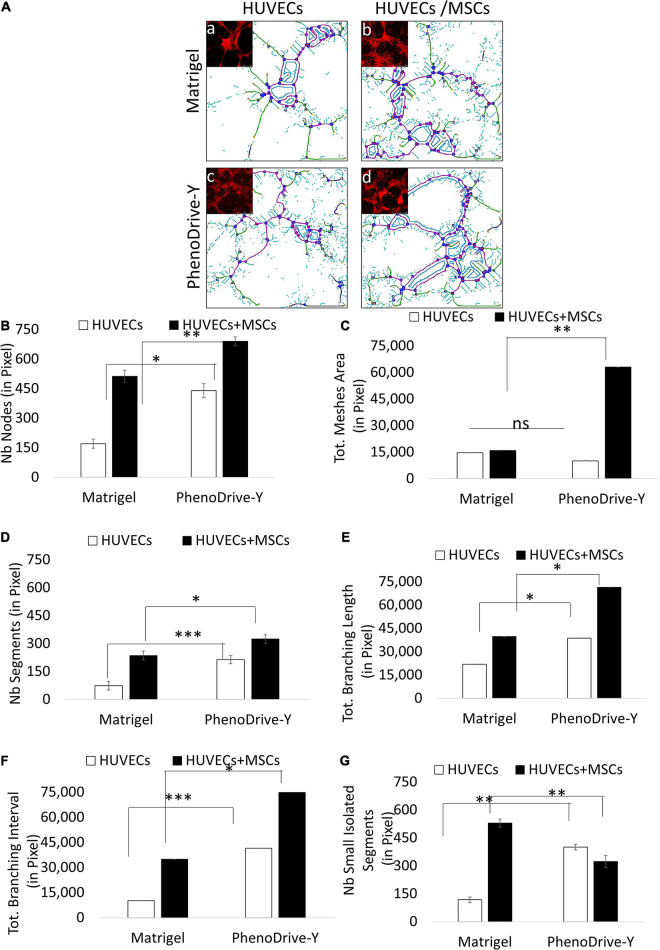
Morphometric analysis of the capillary-like human umbilical vascular endothelial cell (HUVEC) tubules at 48 h. **(A)** Microscopic images analyzed using ImageJ software with the Angiogenesis Analyzer plug-in and identified as nodes in *red circled blue*, segments in *magenta*, small isolated segments in *cyan*, meshes in *blue*, and branches in *green*. **(B–F)** Average number (Nb) of nodes **(B)**, mesh area **(C)**, segments **(D)**, branching length **(E)**, branching interval **(F)**, and small isolated segments **(G)**. ^∗^*p* ≤ 0.01, ^∗∗^*p* ≤ 0.001, ^∗∗∗^*p* < 0.0001 (± SD; *n* = 9). (*a*–*d*) Phalloidin–rhodamine staining (*red*) *Scale bar*, 500 μm.

The cell spheroidal clusters were identified as MSCs by a typical stem cell marker, CD90^+^ (Figure 2A, green-stained cells). More specifically, in Matrigel, these cells were loosely organized around the EC sprouting (Figure 2A, top micrograph, green arrows), unlike the co-cultures on PhenoDrive-Y where compact spheroids were established prevalently at the anastomotic junctions and seemed to stem from the wall of the tubular endothelial structures (Figure 1A, bottom micrograph, red arrows). These results were linked to the epigenetic regulation of the transcriptional factor H3K27me3, known to be critical for MSC stemness, which seemed to be stimulated by the close contact of MSCs with HUVECs (Wu and Lian, 2017). Indeed, the level of expression of H3K27me3 was found to be higher within MSCs’ spheroids growing in close contact with HUVEC tubules on PhenoDrive-Y after 48 h incubation (Figures 2B,C). The morphological observation was followed by a quantitative analysis of the accepted parameters of angiogenesis with ImageJ plug-in analysis (Figures 3A,G). The analysis revealed that the presence of MSCs supported the consolidation of the angiogenic network, as demonstrated by the increase of the anastomotic features (Figure 3A, and inserts *a*–*d* showing the respective rhodamine–phalloidin staining at lower magnifications) and analyzed as the number of anastomosis (Figure 3B, number of anastomotic nodes) and mesh area (Figure 3C). Likewise, the number of branching emerging from the anastomotic nodes (Figure 3D), their length (Figure 3E), and the total segment length/number of branches (Figure 3F, intervals) were also increased in HUVECs/MSCs on PhenoDrive-Y, with the exception of the number of isolated tubules that was higher in the case of Matrigel (Figure 3G), suggesting superior sprouting properties for this substrate, whilst PhenoDrive-Y appears to promote the consolidation of the microvascular network after 48 h incubation.

The changes observed in both (i) the morphometric features of the co-culture and (ii) the regulation of the MSC stemness marker prompted a closer investigation of the effect of the substrates on the tissue-like formation process.

**FIGURE 4:**
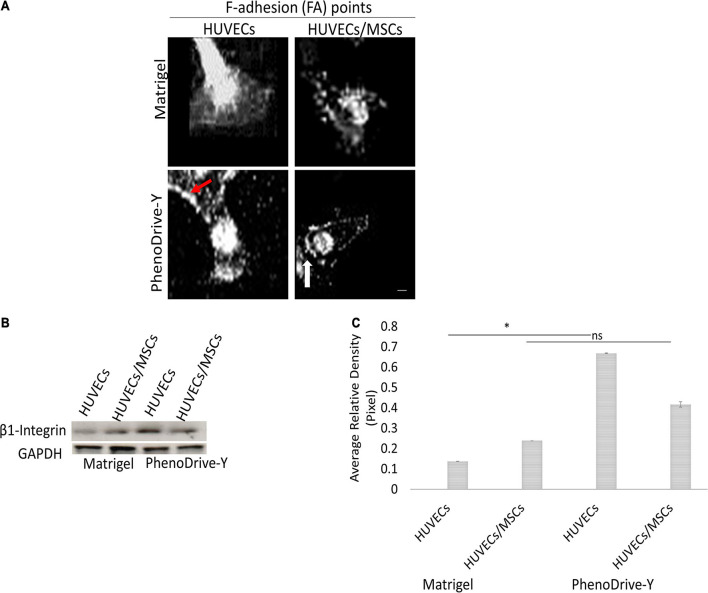
Cytoskeleton formation in monoculture and co-cultured human umbilical vascular endothelial cells (HUVECs) after 48 h incubation. **(A)** Distribution of F-adhesion (FA) points at the HUVEC leading edge (*red arrows*, cluster of focal points; *white arrow*, actin bundles). *Scale bar*, 25 μm. **(B)** Western blot and **(C)** quantification by densitometry of levels of β1 integrin regulation normalized to GAPDH (*n* = 6). *p < 0.01, ns not significant difference.

The process of EC sprouting was studied with rhodamine–phalloidin staining and focused on the behavior of the tip cells of HUVECs on both substrates when in monoculture or in co-culture with MSCs (Figure 4). As suggested by the morphometric analysis, HUVEC tip cells adhering on Matrigel exhibited an elongated shape with a cytoskeleton characterized by stress fiber disjoint from the membrane lamellipodia, which in turn did not show pronounced focal points of adhesion (Figure 4A, Matrigel, HUVECs). The stress fiber of the cytoskeleton appeared to be significantly reduced in the case of HUVECs/MSCs, whilst the formation of a few focal points of adhesion was observed (Figure 4A, Matrigel, HUVECs/MSCs), supporting the observation that the presence of MSCs promotes the stabilization of the formed tubular structures rather than the process of sprouting. A stable anchoring of HUVECs to the substrate appeared more evident on PhenoDrive-Y, where actin was structured into focal points of adhesion at high density along the cell perimeter (Figure 4A, PhenoDrive-Y, HUVECs, red arrow) and with no significant formation of stress fiber. The co-culture with MSCs on this substrate clearly led to the almost complete disappearance of actin fiber and the formation of focal points very regularly spaced in HUVEC tip cells, particularly at the protruding pole of the cell (Figure 4A, PhenoDrive-Y, HUVECs/MSCs). The cell pole interfacing with the existing tubule still maintained a high density of clustered focal points, as shown in Figure 4A (PhenoDrive-Y, HUVECs/MSCs, white arrow). Moreover, Western blot analysis (Figure 4B) and its relative densitometry (Figure 4C) demonstrated that the regulation of β1 integrin in HUVECs cultured on Matrigel was very low and was increased by the presence of MSCs in co-culture. In the case of PhenoDrive-Y, a relatively high expression of β1 integrin was observed in both monoculture and co-culture at 48 h (Figures 4B,C).

**FIGURE 5:**
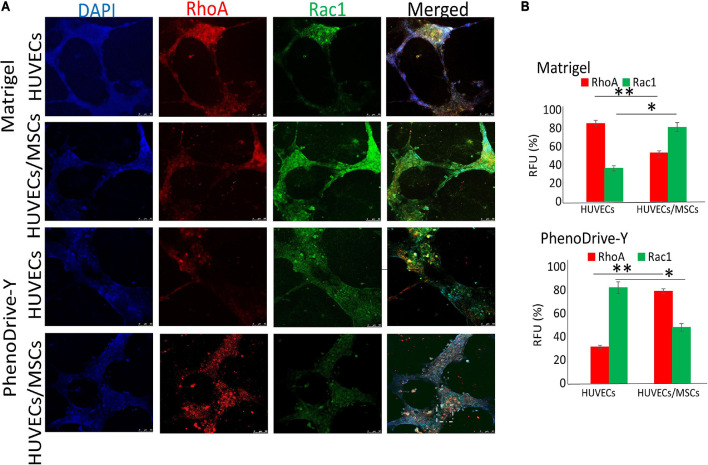
Regulation of RhoA and Rac1 within human umbilical vascular endothelial cells (HUVECs) and HUVECs/mesenchymal stromal cells (MSCs), forming tubules on Matrigel and PhenoDrive-Y after 48 h incubation. **(A)** Staining of RhoA (*red*) and Rac1 (*green*) and cellular nuclei (*blue*). *Dashed circle* represents clusters of cells distributed around the tubules. *Scale bar*, 50 μm. **(B)** Percentage of average fluorescence intensity (RFU) calculated using Fiji ImageJ software. ^∗^*p* ≤ 0.01, ^∗∗^*p* ≤ 0.001 (± SD; *n* = 12).

**FIGURE 6:**
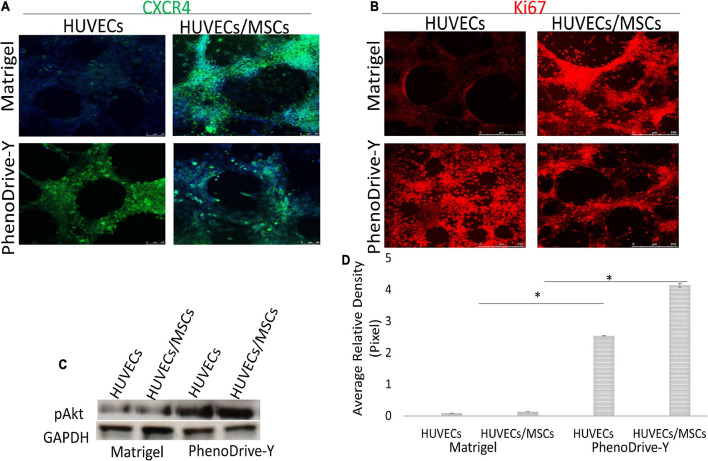
**(A,B)** Localization of markers for CXCR4 (*green*) **(A)** and Ki67 (*red*) **(B)** compared to DAPI nuclear staining (*blue*). *Scale bar*, 50 μm. **(C,D)** pAkt regulation within the monocultured and co-cultured human umbilical vascular endothelial cells (HUVECs) on both matrices at 48 h **(C)** and its quantification **(D)**. *p ≤ 0.01.

The expression and the clustering of integrins into focal adhesion points control the formation of the cell cytoskeleton *via* GTPase activation. Specifically, RhoA-GTPase is known to participate in the regulation of stress fiber in the retracting pole of migrating cells (Chen and Dinh-Xuan, 2014; D’Amico and Mavria, 2010), whilst Rac1-GTPase promotes membrane ruffling (Chen and Dinh-Xuan, 2014; Figures 5A,B). In this study, HUVECs seeded onto Matrigel showed a relatively high RhoA regulation that coincided with a low Rac1 expression mainly localized in the anastomotic areas of the EC capillary networks (Figure 5A, Matrigel, HUVECs). However, the presence of MSCs reverted the RhoA/Rac1 balance whereby Rac1 regulation was higher and uniformly localized across the EC tubules (Figure 5A, Matrigel, HUVECs/MSCs). On PhenoDrive-Y, Rac1 was weakly and uniformly expressed in HUVEC tubule networks (Figure 5A, PhenoDrive-Y, HUVECs), but rare or null within the co-culture (Figure 5A, PhenoDrive-Y, HUVECs/MSCs), whereas the RhoA signal followed the opposite behavior, being more expressed within HUVECs/MSCs and distributed alongside the anastomotic areas of EC tubules (Figure 5A, PhenoDrive-Y, HUVECs/MSCs). These observations were quantitatively confirmed by RFU analysis (as shown in Figure 5B) after 48 h incubation. The data are consistent with previous findings showing that Rac1 becomes an important factor in angiogenesis only when certain types of integrins are not available (D’Amico and Mavria, 2010). CXCR4, an SDF-1 receptor regulating both angiogenesis and stem cell mobilization from their niche (Li et al., 2017), was analyzed to understand the effect of the two substrates on the mechanism of formation of the perivascular stem cell niche. HUVECs sprouting on Matrigel showed no significant expression of this receptor (Figure 6A, Matrigel, HUVECs), which agrees with previous works whereby the level of CXCR4 regulation in ECs varies depending on the type of ECs and their sources, indicating their ability to promote angiogenesis (Gupta and Stadel, 1998). In this study, although SDF-1 spiking was not performed, the lower levels of CXCR4 in HUVECs seeded on Matrigel corroborate the observations of the limited angiogenic properties of this substrate (Figure 1A, Matrigel). Consistent with the previous analyses, HUVECs on PhenoDrive-Y highly stained positive for this marker, suggesting that these cells had acquired a phenotype more prone to establish angiogenic networks (Figure 6A, PhenoDrive-Y, HUVECs).

Unlike HUVECs, MSCs, prevalently located on and around EC anastomotic areas, expressed CXCR4 despite the substrate on which the co-culture was performed (Figure 6A, Matrigel and PhenoDrive-Y, HUVECs/MSCs), indicating the maintained proneness of MSCs to be mobilized upon SDF-1 stimuli, known to be triggered during tissue healing processes (Wan and Ye, 2017). In the case of cells growing on PhenoDrive-Y, the more compact organization of MSC spheroids makes the localization of these receptors more distinct from that of the EC sprouting that appears less visible in the microscopy image due to underlying the plane of focus of the MSCs.

**FIGURE 7:**
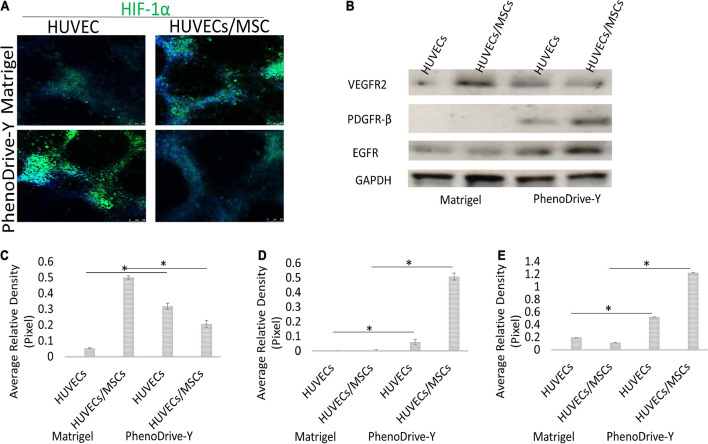
**(A,B)** Expression of HIF-1α **(A)** and specific pro-angiogenic cues **(B)** within cells grown of distinct matrices at 48 h. **(C–E)** The expressions of VEGFR2 **(C)**, PDGFR-β **(D)**, and EGFR **(E)** were assessed and normalized against the GAPDH positive control *p ≤ 0.001.

Likewise, the regulation of the proliferation marker Ki67 was negligible in HUVECs cultured on Matrigel (Figure 6B, Matrigel, HUVECs) compared to those on PhenoDrive-Y (Figure 6B, PhenoDrive-Y, HUVEC). HUVECs/MSCs on both substrates appeared to express high levels of Ki67, suggesting an unaltered renewal potential after 48 h incubation (Figure 6B, Matrigel and PhenoDrive-Y, HUVECs/MSCs). These effects were linked to the activation of the pAkt signaling pathway, which is known to play a key role in EC behavior and, indeed, angiogenesis (Gupta and Stadel, 1998). Immunoblotting analysis showed that the pAkt protein levels were lower in individual and co-cultured cells grown on Matrigel, but significantly elevated in HUVECs and HUVECs/MSCs growing on PhenoDrive-Y (Figures 6C,D). The data indicate a paracrine effect of the MSCs on HUVECs, thus suggesting a wider role played by MSCs on angiogenesis in the perivascular niche (Wan and Ye, 2017). Cells expressed HIF-1α in the anastomotic areas of HUVECs and in the MSC spheroids formed on top of them (Figure 7A). A relatively higher hypoxic status is indeed expected in areas of 3D organization, where oxygen diffusion within the 3D structures is limited (Lee and Sessa, 2014). The hypoxic status *per se* is an angiogenic stimulus (Putnam, 2014) that, in the experiments performed, seemed to be accompanied by the higher expressions of other typical angiogenic factors (Figure 7B). PhenoDrive-Y stimulated the expressions of key receptors for angiogenesis, i.e., VEGFR2 and EGFR, and for PDGF-β (PDGFR-β) that is known to promote the proliferation of pericytes and smooth muscle cells in the blood vessel wall (Figures 7C–E; Baker and Chen, 2012). At 48 h, the expressions of all these receptors on cells cultivated on Matrigel were either undetectable or induced at lower levels. The protein expression levels of VEGFR2 and EGFR were found to be higher compared to those of PDGFR-β, which were increased when HUVECs were co-cultured with MSCs onto PhenoDrive-Y.

## Discussion

The recent discovery that MSCs are organized as spheroids or “mesenspheres” within their niche has led to reconsideration of the use of *in vitro* culture systems for engineering the perivascular niche (Krock et al., 2011). These are termed artificial basement matrices as they aim to recapitulate both the cell-instructive and cell-responsive activities of the native BM (Tallquist et al., 2003; Tietze and Guck, 2019). ECM- and BM-derived products are usually made using natural proteins with biological cues able to control cell phenotypes both *in vitro* and *in vivo* (Cruz-Acuna and Garcia, 2017). However, these biopolymers have limitations, including batch-to-batch variations and costs, that limit their use in cell culture and regenerative medicine (Zhang and Liu, 2011). Recently, PhenoDrive-Y has been shown to drive MSCs into 3D spheroids, where the stemness and paracrine activity of cells are enhanced in comparison to those of the 2D fibroblast-like colonies obtained on plasticware. The effect of this biomimetic biomaterial on MSCs has been demonstrated to be linked to its ability to recapitulate both the main structural and functional properties of the BM, i.e., the mesh-like structure of collagen IV and an ordered spacing of the bioligands present in laminin proteins (Chen and Dinh-Xuan, 2014). These results, together with the reported correlation between angiogenesis and MSCs (Cruz-Acuna and Garcia, 2017), prompted the study of the effect of PhenoDrive-Y on HUVEC/MSC co-culture. In particular, the ability of this biomaterial to drive the formation of endothelial sprouting was investigated in comparison to a substrate widely used for this purpose, Matrigel. The comparative study was performed on both HUVEC monoculture and HUVEC/MSC co-culture to examine the role of BM-mimicking substrates and the effects of cell-to-cell interactions and paracrine signaling. It has been suggested that the MSC-mediated blood vessel formation can be attributed to their ability to either release specific growth factors (e.g., VEGF) or differentiate into ECs (Huang and Feng, 2017). Whilst the latter seems unlikely, there is strong evidence of the paracrine role played by MSCs in the process of angiogenesis and stimuli originating from the perivascular niche (Hagbard and Kallur, 2018). However, these pathways have not yet been completely identified (Huang and Li, 2008). In this study, PhenoDrive-Y was shown to support a pronounced endothelial sprouting and to promote the ordered formation of 3D spheroids of MSCs on top of the anastomotic areas. Unlike Matrigel, where sprouting was relatively reduced and the MSCs more dispersed, co-culture of HUVECs/MSCs on PhenoDrive-Y more closely resembled the formation of a well-structured perivascular MSC niche. The formation of these tissue-like structures had a significant effect on both MSC phenotype and angiogenesis. In terms of phenotype, MSCs were shown to maintain the main characteristics necessary to a functioning niche, i.e., relatively higher stemness, renewal potential, and mobilization predisposition. The paracrine activity necessary to promote angiogenesis was also significantly higher than that on Matrigel, suggesting that, when residing in their niche, MSCs may play a role in ensuring the viability and remodeling of the surrounding blood vessels.

Although Matrigel is known to be made of BM proteins such as collagen and laminin, its lack of an ordered nanotopography and bioligand presentation seems to limit the ability of this substrate to induce the formation of a defined perivascular MSC niche and to stimulate paracrine activities (da Silva Meirelles et al., 2008; Mishra and Ke, 2020). Instead, the results of this and previous studies on PhenoDrive-Y show that this biomaterial can present cells with a mesh-like structure similar to that of a native collagen type IV from which laminin bioligands for β1 integrin cell receptors emerge at an ordered distance. The study shows that an orderly spacing plays a key role in intracellular signals critical to angiogenesis. By driving the organization of actin into orderly spaced focal points rather than stress fiber, PhenoDrive-Y establishes in only 18 h endothelial tubule meshes capable of supporting MSC niches. Previous co-culture on Matrigel have shown that both the sprouting and network formation of HUVECs are strongly reduced due to the initial undefined cell–cell interactions that induce cells to proliferate, migrate, and form tubules after 14 days of incubation or later (Crabtree and Subramanian, 2007). Noticeably, this study highlighted the role that the ordered tissue-like organization plays on the control and linked activation of other key factors of angiogenesis, such as HIF-1 (Adree and Hilfiker, 2019), CXCR4, (Salcedo and Oppenheim, 2003; Smith and Staton, 2006), and Akt (Karar and Maity, 2011; Hsiao and Dilley, 2013). Finally, the expressions of VEGFR2, EGFR, and PDGFR-β supported the reciprocal survival and functional activities played by both HUVECs and MSCs in the perivascular stem cell niche.

## Data Availability Statement

The raw data supporting the conclusions of this article will be made available by the authors, without undue reservation.

## Author Contributions

VP designed the experimental matrix, executed the experimental work, collected, analyzed and interpreted the data, and wrote the manuscript draft. MS originated the research hypothesis, designed the experimental matrix, analyzed and interpreted the data, and reviewed the manuscript. Both authors contributed to the article and approved the submitted version.

## Conflict of Interest

MS is the Director of Tissue Click Ltd., the company commercializing PhenoDrive. The study was performed independently by the research team at the University of Brighton receiving no financial support or any other involvement of the company. The remaining author declares that the research was conducted in the absence of any commercial or financial relationships that could be construed as a potential conflict of interest.

## Publisher’s Note

All claims expressed in this article are solely those of the authors and do not necessarily represent those of their affiliated organizations, or those of the publisher, the editors and the reviewers. Any product that may be evaluated in this article, or claim that may be made by its manufacturer, is not guaranteed or endorsed by the publisher.

## Footnotes

1
http://rsbweb.nih.gov/ij

## Supplementary Material

Supplementary Figure 1 Endothelial sprouting of HUVECs monocultures and HUVECs/MSCs co-cultures after 48 h in different areas of Matrigel- and PhenoDrive-Y-coated wells **(A)**, MSC spheroid formation on Matrigel at 18 and 48 h **(B)**.
