# Supplementary figures and images for: A Substrate-Mimicking Basement Membrane Drives the Organization of Human Mesenchymal Stromal Cells and Endothelial Cells Into Perivascular Niche-Like Structures
Source: Front Cell Dev Biol. 2021 Sep 28;9:701842. doi: 10.3389/fcell.2021.701842 (PMC8507467; doi:10.3389/fcell.2021.701842)

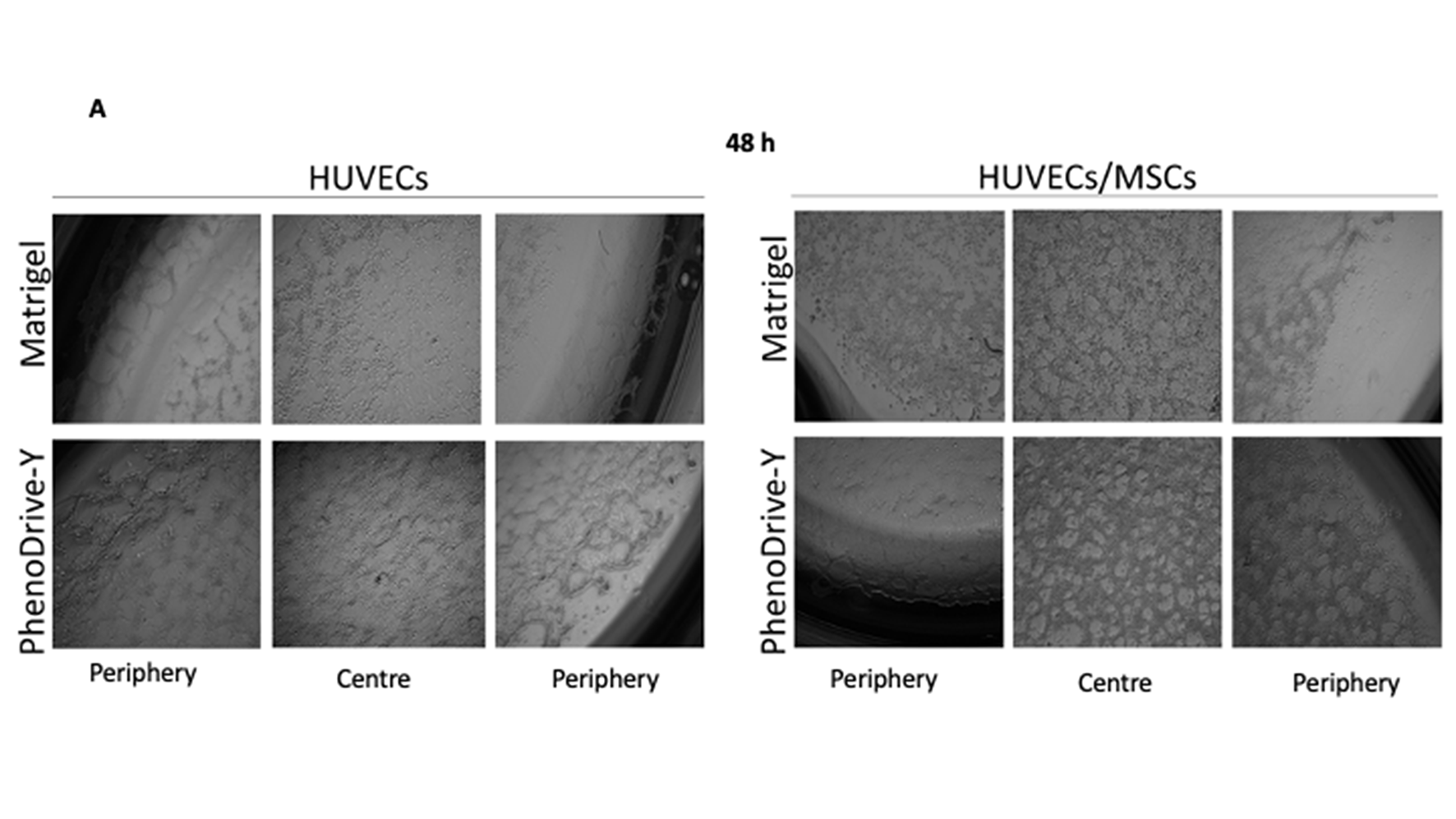

Supplement: Supplementary Figure 1 — Endothelial sprouting of HUVECs monocultures and HUVECs/MSCs co-cultures after 48 h in different areas of Matrigel- and PhenoDrive-Y-coated wells (A), MSC spheroid formation on Matrigel at 18 and 48 h (B). [file Image_1.TIFF]

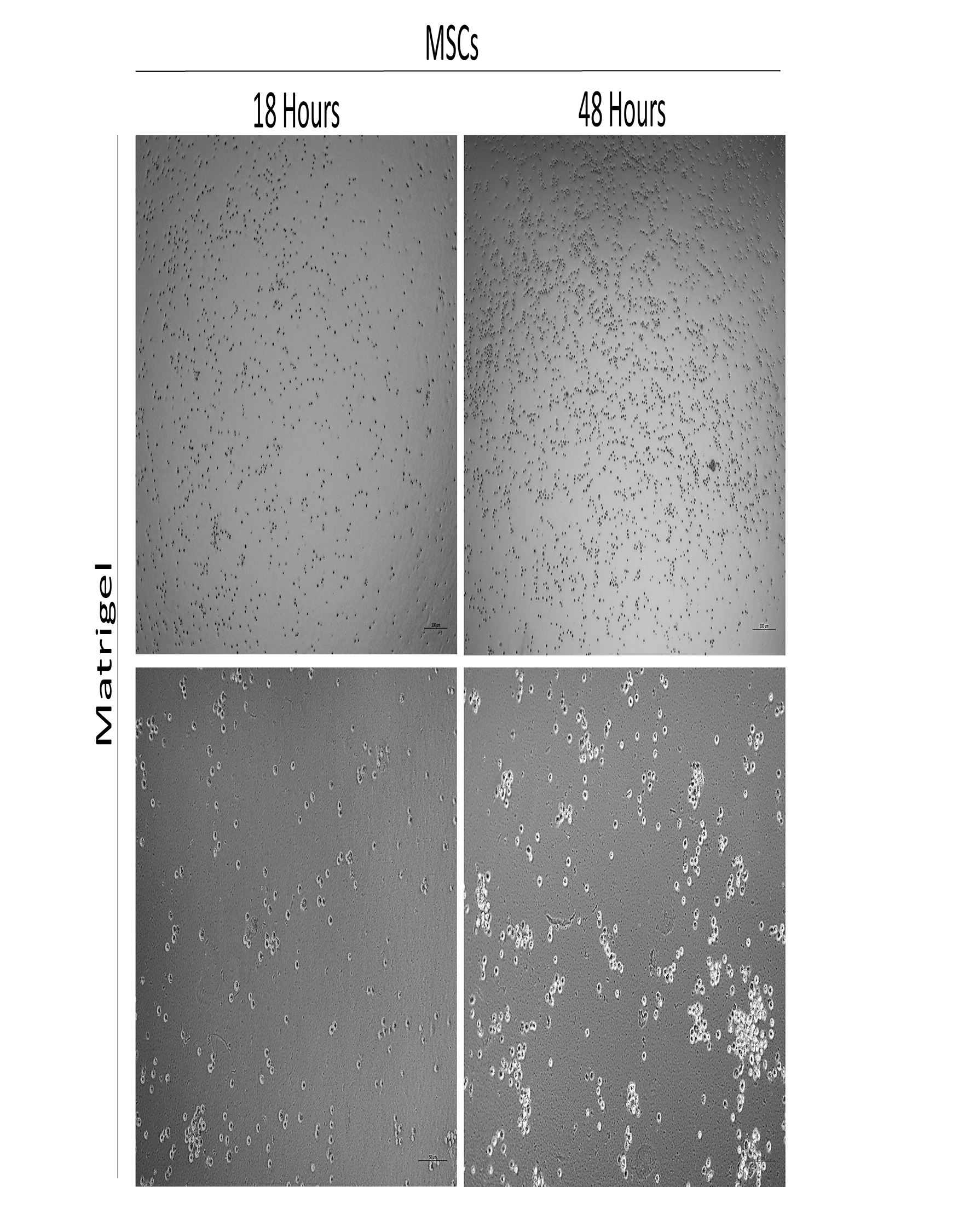

Supplement: Supplementary file 2 [file Image_2.TIFF]
